# *Bartonella* infections in three species of *Microtus*: prevalence and genetic diversity, vertical transmission and the effect of concurrent *Babesia microti* infection on its success

**DOI:** 10.1186/s13071-018-3047-6

**Published:** 2018-08-30

**Authors:** Katarzyna Tołkacz, Mohammed Alsarraf, Maciej Kowalec, Dorota Dwużnik, Maciej Grzybek, Jerzy M. Behnke, Anna Bajer

**Affiliations:** 1 Department of Parasitology, Institute of Zoology, Faculty of Biology, University of Warsaw, 1 Miecznikowa Street, 02-096 Warsaw, Poland; 2 Department of Tropical Parasitology, Institute of Maritime and Tropical Medicine in Gdynia, Medical University of Gdansk, Powstania Styczniowego 9, 81-512 Gdynia, Poland; 3 School of Life Sciences, University of Nottingham, University Park, Nottingham, NG7 2RD UK

**Keywords:** *Babesia microti*, *Bartonella*, Congenital infection, *Microtus*, Poland, Vector-borne zoonotic diseases, Vertical transmission, Voles

## Abstract

**Background:**

*Bartonella* spp. cause persistent bacterial infections in mammals. Although these bacteria are transmitted by blood-feeding arthropods, there is also evidence for vertical transmission in their mammalian hosts. We aimed to determine: (i) the prevalence and diversity of *Bartonella* spp. in a *Microtus* spp. community; (ii) whether vertical transmission occurs from infected female voles to their offspring; (iii) the effect of concurrent *Babesia microti* infection on the success of vertical transmission of *Bartonella*; and (iv) the impact of congenital infection on pup survival.

**Results:**

We sampled 124 *Microtus arvalis*, 76 *Microtus oeconomus* and 17 *Microtus agrestis*. In total, 115 embryos were isolated from 21 pregnant females. In the following year 11 pregnant females were kept until they had given birth and weaned their pups (*n* = 62). Blood smears and PCR targeting the *Bartonella-*specific *rpoB* gene fragment (333bp) were used for the detection of *Bartonella*. *Bartonella* DNA was detected in 66.8% (145/217) of the wild-caught voles. *Bartonella* infection was detected in 81.8% (36/44) of pregnant female voles. *Bartonella*-positive individuals were identified among the embryos (47.1%; 40/85) and in 54.8% (34/62) of pups. Congenitally acquired *Bartonella* infections and co-infection with *B. microti* had no impact on the survival of pups over a 3-week period *post partum*. Among 113 *Bartonella* sequences, four species were detected: *Bartonella taylorii*, *Bartonella grahamii*, *Bartonella doshiae* and a *Bartonella rochalimae*-like genotype. *Bartonella taylorii* clade B was the dominant species in wild-caught voles (49%), pregnant females (47%), their embryos (85%), dams (75%) and pups (95%).

**Conclusions:**

High prevalence of *Bartonella* spp. infection maintained in *Microtus* spp. community is followed by a high rate of vertical transmission of several rodent species of *Bartonella* in three species of naturally infected voles, *M. arvalis*, *M. oeconomus* and *M. agrestis*. Congenitally acquired *Bartonella* infection does not affect the survival of pups. Co-infection with *B. microti* does not affect the effectiveness of the vertical transmission of *Bartonella* in voles. *Bartonella taylorii* clade B was found to be the dominant species in wild-caught voles, including pregnant females and dams, and in their offspring, and was also found to be the most successful in vertical transmission.

**Electronic supplementary material:**

The online version of this article (10.1186/s13071-018-3047-6) contains supplementary material, which is available to authorized users.

## Background

Bacteria of the genus *Bartonella* cause persistent infections in erythrocytes and endothelial cells of mammalian hosts. Infection may contribute to the development of an important vector-borne disease: bartonellosis [1, 2]. Of the 36 named and about 20 “*Candidatus*” species, 17 are recognized as causes of increasing numbers of human and animal bartonellosis cases [2]. Because bartonellosis is characterized by persistent intravascular infection, serious disease syndromes may develop, including endocarditis, myocarditis and a range of vascular pathologies [2]. During the last ten years, the number of confirmed *Bartonella*-derived endocarditis cases diagnosed in humans has increased, attributable in some measure to the increasing awareness of these bacteria by medical practitioners [3, 4].

Zoonotic bartonellosis in humans is associated with several species infecting cats and dogs (i.e. *Bartonella henselae*, *Bartonella clarridgeiae*, *Bartonella rochalimae*, *Bartonella vinsonii berkhoffi*) but also with several species (i.e. *Bartonella doshiae*, *Bartonella elizabethae*, *Bartonella grahamii*) for which different rodent species constitute the main hosts [5–7]. Many rodent species are important reservoir hosts of these bacteria, including mice (*Apodemus* and *Peromyscus* spp.) and voles (*Myodes* and *Microtus* spp.) [1, 7–11]. Prevalence of *Bartonella* infections in rodents differs, but may reach 60–70% or even 90% in susceptible host species [10, 12–21]. About 25 rodent-associated *Bartonella* spp. and genotypes have been described to date [5, 7, 22] and this number is constantly increasing. Although these bacteria are transmitted by a range of blood-feeding arthropods, fleas are considered to be the main vectors among rodents [2, 7, 12, 23–26].

There is also evidence for efficient vertical transmission of bartonellae in different rodents. The first study on vertical transmission was conducted on naturally infected cotton rats (*Sigmodon hispidus*) and deer mice (*Peromyscus leucopus*) in the USA [27], and this established that this mode of transmission was possible and highly successful. Vertical transmission of *Bartonella birtlesi* has been experimentally demonstrated in BALB/c mice in which 76% of fetal resorptions were culture positive for *B. birtlesi*, and vascular lesions were observed in the maternal placenta [28]. However, no bacteria were isolated from 58 viable pups born from infected mice in this study [28]. Recently, *Bartonella* DNA was detected in 69% of fetuses of *Bartonella*-infected wood mice (*Apodemus sylvaticus*) from the Barcelona region in Spain [29] and in one of 15 pups born to experimentally infected jirds [26]. In contrast to those studies, no congenital infections were recognized in bank voles *Myodes glareolus* in the UK [12]. Several cases of human congenital bartonellosis have been reported and attributed to different species of *Bartonella* [30, 31], including the case of a 22-day-old boy from Peru [32]. All of the above findings indicate that further studies on vertical transmission of these vector-borne bacteria are still needed to enable a comprehensive epidemiological assessment of the risk of infection by this route.

In our previous studies in rodent hosts, we observed an interesting pattern of host age-related prevalence suggesting the existence of vertical transmission [13, 14, 33, 34]. In contrast to the expectation that the acquisition of vector-borne pathogens should increase with host age as a consequence of the associated increased risk of vector contact with increasing age, we often observed a reversed pattern, with the prevalence of *Bartonella* infection decreasing with host age, and this could not be explained simply by greater exposure of juveniles to ectoparasites. Our aim was to describe the *Bartonella* spp. infection and the possibility of vertical transmission of this parasite in the population of wild-living voles in Poland. We aimed to determine: (i) the prevalence and diversity of *Bartonella* spp. in a *Microtus* spp. community; (ii) whether vertical transmission occurs from infected female voles to their offspring; (iii) the effect of concurrent *Babesia microti* infection on the success of vertical transmission of *Bartonella*; and (iv) the impact of congenital infection on pup survival.

To achieve the aims, we determined the presence of *Bartonella* in embryos dissected from naturally infected voles, since this should completely eliminate the possibility of vector-borne transmission to the embryos. To eliminate the possibility that the tissues of the embryos may have been contaminated by maternal blood, we also maintained in captivity naturally infected pregnant female voles, completely deprived of ectoparasites, until a suitable period after parturition when individual sampling of the blood of the pups was possible. Thus we assessed the prevalence of congenitally transmitted *Bartonella* infection in the pups and evaluated the impact of congenital infection on pup survival. Finally, we identified the bacterial species infecting wild-caught voles and offspring through their distinct molecular signatures. The obtained results expanded the existing knowledge on the prevalence, vertical transmission and species composition of *Bartonella* in wild living rodents in Poland.

## Methods

Field studies were conducted in the Mazury Lake District of north-eastern Poland (Urwitałt, near Mikołajki; 53°48'50.25"N, 21°39'7.17"E), within an extensive forest and old field system adjacent to Lakes Śniardwy and Łuknajno. A detailed description of trapping sites is provided in Tolkacz et al. [35]. In short, three species of voles were live-trapped in different microhabitats extending up gentle hills (greatest elevation 5 m) from two small ponds, giving enough height difference for a gradation in physical conditions and vegetation: from marshland (submerged during rainy weather; optimal habitat for the root vole, *Microtus oeconomus*) to dry grassland (preferable habitat of *Microtus arvalis*). Individuals of *Microtus agrestis* were trapped in the intermediate zones. Trapping took place in summer (August and early September) in 2013 and 2014. Traps were set for at least 5 consecutive nights. All animals (*n* = 217; for details see Table 1 in [35]) were transported in their traps to the laboratory for inspection.

**Table 1:** Prevalence of *Bartonella* spp. in three species of wild-caught *Microtus* voles. Numbers of pregnant females are shown in parentheses; % of pregnant infected females are shown in square brackets

Year	Infection status	*M. arvalis*			*M. agrestis*			*M. oeconomus*			*Microtus* spp.		
		♂	♀	All	♂	♀	All	♂	♀	All	♂	♀	Total
2013	NI	8	8 (5)	16	4	2 (2)	6	4	1 (1)	5	16	11 (8)	27
	I	12	27 (16)	39	5	3 (1)	8	8	6 (4)	14	25	36 (21)	61
	% infected	60.0	77.1 [76.2]	70.9	55.6	60.0 [33.3]	57.1	66.7	85.7 [80.0]	73.7	61.0	76.6 [72.4]	69.3
2014	NI	12	6 (0)	18	1	0 (0)	1	12	14 (0)	26	25	20 (0)	45
	I	21	30 (10)	51	0	2 (0)	2	13	18 (5)	31	34	50 (15)	84
	% infected	63.6	83.3 [100.0]	73.9	0.0	100.0 [–]	66.7	52.0	56.3 [100.0]	54.4	57.6	71.4 [100.0]	65.1
Total	NI	20	14 (5)	34	5	2 (2)	7	16	15 (1)	31	41	31 (8)	72
	I	33	57 (26)	90	5	5 (1)	10	21	24 (9)	45	59	86 (36)	145
	% infected	62.3	80.3 [83.9]	72.6	50.0	71.4 [33.3]	58.8	56.8	61.5 [90.0]	59.2	59.0	73.5 [81.8]	66.8

*Abbreviations*: NI, number of uninfected voles; I, number of infected voles

In 2013, the autopsies were carried out under terminal anaesthesia [35]. Voles were allocated to three age classes (juveniles, young adults and adults) based on body weight and nose-to-anus length together with reproductive condition (scrotal, semi-scrotal or non-scrotal for males; lactating, pregnant or receptive for females) [33]. Ectoparasites were removed and preserved in 99.8% methanol. A blood sample was taken from the heart (for direct preparation of two thin blood smears and storage in 0.001M EDTA for subsequent DNA extraction). Identification of the vole species was performed as described previously [35]. The upper (maxilla) and lower (mandible) jawbones of autopsied individuals were inspected to confirm the identity of the vole species based on the known dental formula for each, and especially to distinguish between juvenile individuals of *M. oeconomus* and *M. agrestis* [36]. We confirmed the species identity of each by examination of the lower molars M_1_ and M_2_ and the second upper molar (M^2^) [36]. Embryos were isolated from the uterus, washed in sterile water and frozen at a temperature of -20 °C.

In the summer of 2014, all captured voles were live-processed as described in Tolkacz et al. [35], including the removal of ectoparasites from anesthetized animals. A blood sample was taken from the tail tip of each vole (for blood smears, preservation in EDTA and DNA extraction). Then, males and juveniles were released near to their trapping points. Females suspected of being pregnant were transferred to individual clean sterile cages to establish a breeding colony of voles [35]. To prevent the development of ectoparasites (i.e. development of nymphs from engorged tick larvae), and to provide suitable housing conditions for voles, the cages were cleaned at least once per week. During handling, all voles from the breeding colony were inspected for ectoparasites in order to ensure vector-free conditions in the cages and animal house. No ectoparasites were noted at any time after initial caging. Females were kept at a constant temperature of 18 °C, and with a 16 (day): 8 (night) light-dark photoperiod for at least 3 weeks to allow pregnancies to develop to term. Non-pregnant females were then released at their original trap lines. Pups were kept together with their dams for one month. In the third week of life we weighed the pups and collected blood samples from the tail tip. Pups and dams were then released at the trap lines at which the dams had been originally caught.

### Blood collection and DNA extraction

**Fig. 1:**
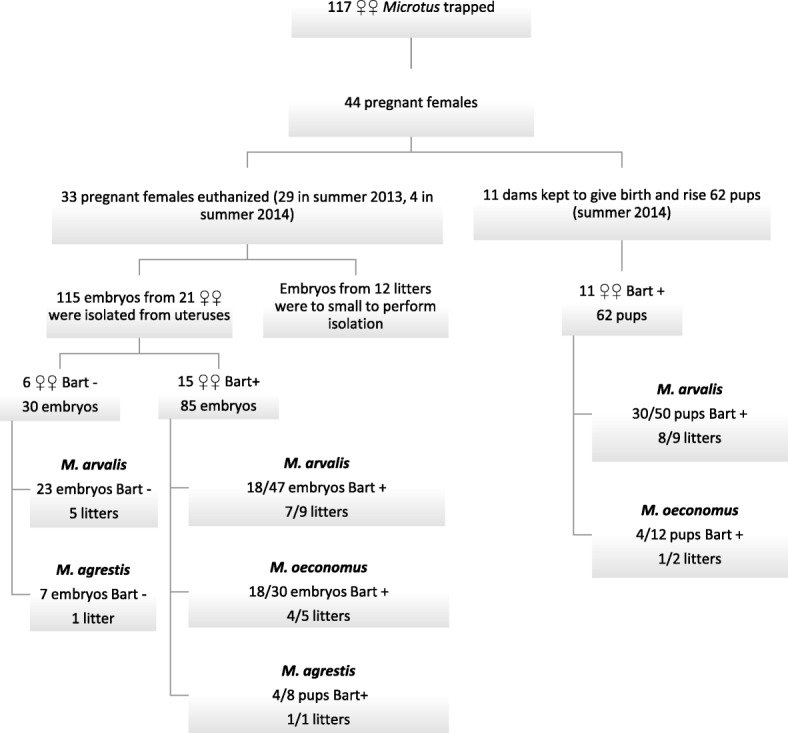
The study design. *Abbreviations*: Bart+, voles infected with *Bartonella* spp.; Bart-, voles uninfected with *Bartonella* spp. (177 embryos and pups in total)

Two blood smears were prepared from wild-caught voles and pups. Smears were air-dried, fixed in absolute methanol and stained with Diff Quick (Microptic SL, Barcelona, Spain) and Hemacolor (Merck, Darmstadt, Germany) staining kits, according to each manufacturer’s instructions. Specific amplification of *Bartonella* DNA was used for the identification of infection in all adult voles (males and females), embryos and pups. Between 20 (from the live-processed voles) and 200 μl (from the culled individuals) of whole blood were collected into 0.001 M EDTA and frozen at a temperature of -20 °C before DNA extraction. Embryos were isolated from the uterus and individually autopsied following two washes in sterile water, to minimize contamination with maternal blood. We autopsied 115 embryos from 21 litters (17 obtained in 2013 and 4 obtained in 2014 from females that succumbed under anaesthesia) (Fig. 1). Hearts and lungs were isolated from embryos with sterile dissecting instruments. Genomic DNA was extracted from whole blood and organs using the DNAeasy Blood & Tissue kit (Qiagen, Hilden, Germany) and stored at a temperature of -20 °C. The remaining 12 litters were too small to enable the isolation of specific internal organs [35].

### Microscopic examination

Smears from all captured animals and pups were examined under oil immersion (×1000 magnification) for parasitaemia estimation. A total of 200 fields of vision were scanned by experienced researchers and the number of infected RBC (iRBC) was recorded. Parasitaemia was expressed as the mean number of iRBC/200 fields of vision.

### Molecular characterization

The detection and genotyping of *Bartonella* species/variants were performed by amplification and sequencing of the 333 bp gene fragment of RNA polymerase β-subunit (*rpoB*) obtained in a single-step PCR or in a nested PCR (in the case of no or weak signal from the initial one-step PCR). The primers and thermal profile used in this study have been described previously [15, 37].

In the first step of the nested PCR, the 825 bp gene fragment of RNA polymerase β-subunit (*rpoB*) was amplified with specific primers: 1400F (5'-CGC ATT GGC TTA CTT CGT ATG-3') and 2300R (5'-GTA GAC TGA TTA GAA CGC TG-3') [37]. The PCR conditions included: 95°C for 5 min, followed by 38 cycles of denaturation at 95 °C for 45 s, annealing at 55 °C for 45 s, and elongation at 72 °C for 45 s. Final elongation was at 72 °C for 7 min, followed by a hold step at 4 °C. The PCR reaction was performed in 1× PCR buffer, 1U Taq polymerase, 1 μM of each primer and 2 μl of the extracted DNA sample. Negative controls were performed in the absence of template DNA.

In the second step (nested reaction) and in the single-step PCR, the forward primer rpoF (5'-GCA CGA TT(C/T) GCA TCA TCA TTT TCC-3') and the reverse primer rpoR (5'-CGC ATT ATG GTC GTA TTT GTC C-3') were used [15]. The PCR conditions included: 95 °C for 5 min, followed by 39 cycles of denaturation at 95 °C for 45 s, annealing at 55 °C for 45 s, and elongation at 72 °C for 45 s. Final elongation was at 72 °C for 7 min, followed by a hold step at 4 °C. The PCR reaction was performed in 1× PCR buffer, 1U Taq polymerase, 1 μM of each primer and 2 μl of the extracted DNA sample in the case of the single PCR. Nested PCR reactions were performed with different volumes of the first PCR product: 1 or 0.5 μl, or finally with 2 μl of a 1:9 dilution in sterile water. In some cases, dilution of the first reaction product improved the output of nested reaction and the intensity of the bands. As positive controls, we used the genomic DNA of *Bartonella acomydis* extracted from wild rodents from Egypt [22].

PCR products were subjected to electrophoresis on a 1.5% agarose gel, stained with Midori Green stain (Nippon Genetics GmbH, Düren, Germany). Selected PCR products from voles trapped in 2013 and 2014, all pregnant females and dams, and at least two embryos/pups per litter (if available), were sequenced by a private company (Genomed S.A., Warsaw, Poland). DNA sequence alignments and analyses were conducted using MEGA v.7.0. Consensus sequences were compared with sequences deposited in the GenBank database (http://www.ncbi.nlm.nih.gov/genbank/).

### Statistical analysis

The statistical approach adopted has been documented comprehensively in our earlier publications [35, 38–40]. Prevalence (percentage of animals infected) was analyzed by maximum likelihood techniques based on log-linear analysis of contingency tables (in SPSS v. 21). The results are presented as percentages with 95% confidence interval (CI), calculated with bespoke software based on the tables of Rohlf & Sokal [41], by courtesy of F. S. Gilbert and J. M. Behnke from the University of Nottingham, UK. For analysis of the prevalence of *Bartonella* in wild-caught voles, we fitted prevalence of *Bartonella* infection as a binary factor and then year (two levels: 2013, 2014), host species (three levels: *M. arvalis*, *M. oeconomus*, *M. agrestis*), host age (three levels: juvenile, young adult, adult) and host sex (two levels: males and females) as factors. Subsequent analyses were carried out for each host species separately.

For analysis of the prevalence of *Bartonella* in embryos, we implemented ‘female infection’ as a binary factor (i.e. infected/uninfected mother). For analysis of the prevalence of *Bartonella* in pups, we implemented pup survival as a binary factor (dead = 0 or alive = 1 at the age of 3 weeks). In order to test the hypothesis that co-infection with *Babesia microti* in females/dams may facilitate congenital transmission of *Bartonella* to their embryos/pups, we fitted models with *B. microti* infection of female/dam and embryo/pup as an additional factor (coded as infected = 1, uninfected = 0). Beginning with the most complex model, involving all possible main effects and interactions, those combinations not contributing significantly to the explanation of variation in the data were eliminated stepwise, beginning with the highest-level interaction. A minimum sufficient model was then obtained, for which the likelihood ratio of *χ*^2^ was not significant, indicating that the model was sufficient in explaining the data.

General linear models (GLMs in SPSS v.21) were used for comparison of mean parameters (abundance of *Bartonella*, litter size, mean weight of pups, etc.), which are reported with standard errors of their means (SE). The abundance of *Bartonella* infection was calculated as the number of infected red blood cells (iRBC) in 200 fields of vision (×1000 magnification). When samples were only positive by PCR, an intensity of 0.001 iRBC/200 fields was implemented into the quantitative statistical analysis.

The success of vertical transmission to each litter, calculated as the fraction of *Bartonella*-positive pups/litter was correlated with the litter size using the Spearman’s rank correlation test (SPSS v. 21). Fisher’s exact test (INSTAT software) was used to compare the percentage of infected pups between *Bartonella*-negative and *Bartonella*-positive females.

## Results

### Prevalence of *Bartonella* spp. in the community of voles

In total, 217 voles of three species were trapped and sampled: 124 common voles, *M. arvalis*; 76 root voles, *M. oeconomus*; and 17 field voles, *M. agrestis*. Prevalence of *Bartonella* spp. infection by year of study, host species and sex is provided in Table 1. Prevalence was calculated by the PCR results and microscopical observation of blood smears. As these methods do not allow different bacterial species to be distinguished, we refer to ‘*Bartonella* spp.’ infection. In total, a positive product of the PCR reaction and microscopy was obtained for 66.8% (95% CI: 62.4–71.0%) of voles in the community. The highest prevalence of *Bartonella* spp. was detected in *M. arvalis* (72.6%; 95% CI: 65.2–79.0%), lower and similar in *M. oeconomus* (59.2%; 95% CI: 47.1–70.6%) and in *M. agrestis* (58.8%; 95% CI: 35.0–80.4%), thus prevalence did not vary significantly between the three host species (*Bartonella* infection × host species: *χ*^2^_2_ = 3.72, *P* = 0.156) (Table 1). Although minor differences in prevalence of *Bartonella* spp. between the two years of the study (Table 1) were observed, they were not significant (ns). However, prevalence of *Bartonella* spp. was higher in female voles (*Bartonella* infection × host sex: *χ*^2^_1_ = 5.12, *P* = 0.024; Table 1).

Age-related effects on prevalence differed among host species (*Bartonella* infection × host species × age class: *χ*^2^_4_ = 15.00, *P* = 0.007). In *M. arvalis*, prevalence was high in juvenile voles (12/16, 75%; 95% CI: 50.0–91.0%), highest in young adults (19/19, 100%; 95% CI: 82.5–100.0%) but lowest in the oldest adults (59/89, 66.3%; 95% CI: 53.2–77.7%) (*Bartonella* infection × age class: *χ*^2^_2_ = 13.92, *P* = 0.001). In *M. oeconomus*, prevalence increased gradually with host age (40.0%, 57.0% and 63.5% in age class 1, 2 and 3, respectively) but the difference in prevalence between these age classes was not significant. In *M. agrestis*, the effect of age was not significant as our sample only included one juvenile individual of this species, which was found to be infected (100%). Prevalence was 50% in young adults and 60% in adult field voles (ns).

### Abundance of *Bartonella* spp. infection in the community of voles

Mean abundance of *Bartonella* infection by year of study, host species and sex is provided in Table 2. The mean abundance of *Bartonella* infection, calculated for the three vole species combined, was low: 2.58 ± 2.06 iRBC/200 fields of vision.

**Table 2:** Abundance of *Bartonella* spp. in wild-caught voles. Mean number of infected red blood cells (iRBC)/200 fields of vision ± SE

Species	Year		
	2013	2014	Total
*Microtus arvalis*			
Males (*n* = 53)	9.06 ± 9.01	2.28 ± 8.02	5.67 ± 6.03
Females (*n* = 71)	7.23 ± 4.23	6.06 ± 4.87	6.64 ± 3.22
Combined sexes (*n* = 124)	8.14 ± 4.98	4.17 ± 4.69	6.16 ± 3.42
*Microtus oeconomus*			
Males (*n* = 37)	0.55 ± 1.5	0.11 ± 1.08	0.28 ± 0.89
Females (*n* = 39)	0.11 ± 1.27	0.87 ± 0.80	0.49 ± 0.75
Combined sexes (*n* = 76)	0.29 ± 0.98	0.49 ± 0.67	0.40 ± 0.58
*Microtus agrestis*			
Males (*n* = 10)	3.14 ± 3.73	0.00 ± 9.30	2.10 ± 3.98
Females (*n* = 7)	0.00 ± 4.39	0.00 ± 6.58	0.00 ± 3.68
Combined sexes (*n* = 17)	1.26 ± 3.03	0.00 ± 5.70	0.90 ± 2.71
*Microtus* spp.			
Males (*n* = 100)	4.94 ± 4.40	1.02 ± 4.50	2.97 ± 3.15
Females (*n* = 117)	2.45 ± 3.78	2.97 ± 3.08	2.20 ± 2.65
Combined sexes (*n* = 217)	3.83 ± 2.16	1.34 ± 3.51	2.58 ± 2.06

Although there were some differences in the mean abundance of *Bartonella* spp. between host species, years of study, age classes and sexes, none of these factors affected abundance significantly. Numerically, abundance was highest among *M. arvalis*, lower in *M. agrestis* and the lowest in *M. oeconomus*, as observed for prevalence but the difference in abundance between host species was not significant (main effect of host species on abundance: *F*_(2, 216) _= 2.2, *P* = 0.114) (Table 2).

Similarly, host age did not have a significant effect on abundance, although the mean values for age classes followed the same sequence as for prevalence among *M. arvalis* and *M. oeconomus*: abundance of *Bartonella* was highest in age class 2 (young adults) and lowest in juvenile voles. In *M. agrestis*, we observed highest abundance in adult individuals and lowest in juveniles and young adults.

### Vertical transmission of *Bartonella*

#### *Bartonella* spp. infection in females and dams

Altogether 117 female voles were trapped, among which 44 were pregnant thus providing 26 litters (embryos and pups) from *Bartonella*-positive females. We excluded 12 litters because pregnancy was still at a very early stage and the embryos were too small to enable reliable isolation of fetal tissues. Six litters from *Bartonella*-negative mothers were available as a control group for analysis of vertical transmission (Fig. 1). The overall prevalence of *Bartonella* infection in the pregnant females was 81.8% (95% CI: 65.2–91.6%) (Table 1). The highest prevalence was noted in pregnant *M. oeconomus* voles (90%; 95% CI: 55.4–99.5%), then 83.9% (95% CI: 70.8–92.4%) in *M. arvalis* females, but only one female of three pregnant *M. agrestis* (33.3%; 95% CI: 1.7–86.5%) was found to be *Bartonella*-positive (Table 1, Fig. 1).

We were able to analyze the prevalence of infections in 115 embryos from 21 litters (including 15 litters from *Bartonella*-positive females and 6 litters from *Bartonella*-negative females; Fig. 1, Table 3).

**Table 3:** Evidence for vertical transmission and genotype identity of *Bartonella* spp. in embryos isolated from female voles in 2013 and 2014

ID of pregnant female	Host species	No. of embryos in litter	No. of embryos infected with *Bartonella* spp. in litter	% of infected embryos	*Bartonella* strain	
					In positive female	No. of genotyped embryos
2013/3	*M. arvalis*	7	1	14.3	nd	1× *B. taylorii* (clade B)
2013/21	*M. arvalis*	6	4	66.7	*B. taylorii* (clade B) + *B. rochalimae*-like	2*× B. rochalimae-*like + *B. taylorii* (clade B); 1× *B. taylorii* (clade B)
2013/36	*M. agrestis*	8	4	50.0	*B. taylorii* (clade B)	3× *B. taylorii* (clade B)
2013/37	*M. arvalis*	5	3	60.0	*B. taylorii* (clade B)	3× *B. taylorii* (clade B)
2013/41	*M. arvalis*	6	2	33.3	*B. taylorii* (clade A)	1× *B. taylorii* (clade B)
2013/45	*M. arvalis*	2	1	50.0	*B. grahamii*	1× *B. taylorii* (clade B)
2013/63	*M. oeconomus*	5	0	0	*B. taylorii* (clade B)	nd
2013/70	*M. oeconomus*	6	1	16.7	*B. grahamii*	1× *B. taylorii* (clade B)
2013/72	*M. arvalis*	6	0	0	*B. taylorii* (clade A)	nd
2013/78	*M. arvalis*	7	0	0	*B. taylorii* (clade B)	nd
2013/80	*M. arvalis*	4	3	75.0	*B. taylorii* (clade B) + *B. grahamii*	3× *B. taylorii* (clade B)
2014/19	*M. oeconomus*	7	7	100	*B. grahamii*	2× *B. grahamii*
2014/44	*M. oeconomus*	6	6	100	*B. grahamii*	nd*
2014/68	*M. oeconomus*	6	4	66.7	*B. taylorii* (clade B) + *B. doshiae*	4× *B. taylorii* (clade B)
2014/155	*M. arvalis*	4	4	100	nd	3× *B. taylorii* (clade B)
Total	15 (9 *Ma*; 5 *Mo*; 1 *Mag*)	85 (47 *Ma*;30 *Mo*; 8 *Mag*)	40 (18 *Ma*; 18 *Mo*; 4 *Mag*)	47.1% (38.3% in *Ma*; 60.0% in *Mo*; 50.0% in *Mag*)	16 *Bartonella* spp. (2 *B. taylorii* clade A; 7 *B. taylorii* clade B; 5 *B. grahamii*; 1 *B. doshiae*; 1 *B. rochalimae-*like)	27 *Bartonella* spp. (23 *B. taylorii* clade B; 2 *B. grahamii*; 2 *B. rochalimae-*like)

*Abbreviations*: nd, not done; nd*, not done because of a weak signal; *Ma*, *Microtus arvalis*; *Mo*, *Microtus oeconomus*; *Mag*, *Microtus agrestis*

Another 11 *Bartonella*-positive females were kept in captivity until parturition (host species and litter size are provided in Table 4).

**Table 4:** Evidence for vertical transmission and genotypes of *B. taylorii* in pups delivered by female voles captured in 2014

ID of pregnant female	Host species	No. of pups in litter	No. of pups infected with *Bartonella* spp. in litter	% of infected pups	*Bartonella* strain	
					In positive dams	No. of genotyped pups
2014/25	*M. arvalis*	6	5	83.3	nd	3× *B. taylorii* (clade B)
2014/34	*M. arvalis*	5	5	100	*B. taylorii* (clade B)	3× *B. taylorii* (clade B)
2014/59	*M. arvalis*	5	5	100	nd	3× *B. taylorii* (clade B)
2014/65	*M. arvalis*	6	6	100	*B. taylorii* (clade B)	3× *B. taylorii* (clade B)
2014/74^a^	*M. oeconomus*	6	0	0	*B. taylorii* (clade B)	nd
2014/77	*M. oeconomus*	6	4	66.7	nd	3× *B. taylorii* (clade B)
2014/107^a^	*M. arvalis*	6	6	100	*B. taylorii* (clade B)	3× *B. taylorii* (clade B)
2014/112	*M. arvalis*	5	0	0	*B. taylorii* (clade A)	nd
2014/126	*M. arvalis*	7	1	14.3	*B. taylorii* (clade A)	1× *B. taylorii* (clade A)
2014/130	*M. arvalis*	4	1	25	*B. taylorii* (clade B)	1× *B. taylorii* (clade B)
2014/131	*M. arvalis*	6	1	16.7	*B. taylorii* (clade B)	1× *B. taylorii* (clade B)
Total	11 (9 *Ma*; 2 *Mo*)	62 (50 *Ma*; 12 *Mo*)	34 (30 *Ma*; 4 *Mo*)	54.8% (60% in *Ma*; 33% in *Mo*)	2 *B. taylorii* clade A (2 *Ma*); 6 *B. taylorii* clade B (5 *Ma*+1 *Mo*)	1 *B. taylorii* clade A (1 *Ma*); 20 *B. taylorii* clade B (18 *Ma* + 3 *Mo*)

^a^Pups died after birth

*Abbreviations*: nd, not done; *Ma*, *Microtus arvalis*; *Mo*, *Microtus oeconomus*

#### Detection of *Bartonella* in pregnant females and embryos (2013 and 2014)

Prevalence of *Bartonella* spp. infections, as determined by PCR among the 115 embryos of the 21 females, was 34.8% (95% CI: 28.0–42.2%). No *Bartonella* DNA was detected in 30 embryos of the six *Bartonella*-negative females (5 *M. arvalis* and 1 *M. agrestis*), in comparison to 47.1% (40/85; 95% CI: 34.5–59.9%) of positive embryos recovered from 15 *Bartonella*-positive females (Fisher’s exact test: *P* < 0.0001). Among *Bartonella*-positive pregnant females, nine were *M. arvalis*, five *M. oeconomus* and one *M. agrestis* (Fig. 1, Table 3). *Bartonella*-positive embryonic tissues (heart and lungs) were found in 80% (95% CI: 53.4–94.3%) of these litters.

The proportion of litters from *Bartonella* positive females, in which at least one embryo was *Bartonella*-positive, was similar among the three host species (Fig. 1, ns). However, the proportion of *Bartonella*-positive embryos carried by *Bartonella* positive females differed among the three host species (Fig. 1; *χ*^2^_2_ = 11.92, *P* = 0.003). Among nine *Bartonella*-positive females of *M. arvalis*, positive embryos were identified in 77.7% (95% CI: 44.2–95.9%) of litters but the overall prevalence among embryos was 38.3% (95% CI: 22.9–56.0%) (Fig. 1, Table 3). *Bartonella* DNA was detected in 80% (95% CI: 34.3–99.0%) of litters and 60% (95% CI: 41.6–76.4%) of embryos of five *Bartonella*-positive *M. oeconomus*. In the one litter of the *Bartonella*-positive *M. agrestis* female, 50% (95% CI: 19.3–80.7%) of embryos were *Bartonella*-positive. Thus, not all embryos carried by infected females were positive for *Bartonella*, and the success of vertical transmission (fraction of positive embryos) of *Bartonella* spp. differed between litters even within host species: in three litters from infected mothers no positive embryos were identified, in another three all tested embryos were *Bartonella*-positive and prevalence of *Bartonella* spp. was within the range 14–75% for the remaining nine litters.

#### Detection of *Bartonella* in dams and pups maintained in vector-free conditions (2014)

In the second year, *Bartonella* DNA was detected in 54.8% (95% CI: 43.9–65.3%) of pups born to 11 *Bartonella*-positive dams (Fig. 1, Table 4). In two litters from *M. oeconomus* dams, 33.3% (95% CI: 12.3–63.0%) of pups were positive, in comparison to 60% (95% CI: 45.0–72.2%) positive pups from nine *M. arvalis* dams (Table 4, ns).

Among the 9 litters from *M. arvalis* dams, *Bartonella*-positive pups were found in 8 litters (89%; 95% CI: 55.7–99.4%) and among positive litters, the percentage of *Bartonella*-positive pups varied within the range 14–100% (Table 4).

There was no correlation between the proportion of *Bartonella*-positive pups in a litter and litter size (Table 4, ns). There was also no significant difference between male and female pups born from infected dams: 55.2% (16/29; 95% CI: 36.0–72.8%) of males and 57.1% (12/21; 95% CI: 35.4–76.7%) of females were infected with *Bartonella* spp.

The abundance of *Bartonella* spp. was calculated by microscopical observation of blood smears of 44 *M. arvalis* and 6 *M. oeconomus* pups. The mean abundance of *Bartonella* in blood smears collected from offspring of infected dams was 1.61 ± 0.35, but this was more than twice as high in *M. oeconomus* compared with *M. arvalis* pups (2.5 ± 0.67 and 0.72 ± 0.24, respectively; main effect of host species on *Bartonella* abundance: *F*_(1, 49)_ = 6.33, *P* = 0.015).

### Effect of *B. microti* infection on vertical transmission of *Bartonella*

In our previous study [35] on the same samples we provided evidence for a high rate of vertical transmission of *B. microti* in wild voles. In the minimal sufficient model derived from analysis of a model that included *B. microti* infection, only the presence of *Bartonella* infection in females/dams had a significant effect on *Bartonella* infection in embryos/pups (*χ*^2^_1_ = 16.89, *P* < 0.001). *Babesia* infection in females/dams had no effect: the success of vertical transmission of *Bartonella* was 32.4% (12/37; 95% CI: 19.4–47.9%) in embryos/pups of *Babesia*-negative females/dams and 44.3% (62/140; 95% CI: 36.4–52.5%) in embryos/pups of *Babesia*-positive females/dams, and the difference was not significant. Focusing on the infection status of offspring, we correlated the success of vertical transmission of *Babesia* in the litters (fraction of *Babesia*-infected litter) with the success of vertical transmission of *Bartonella* in the litter (fraction of *Bartonella*-infected litter), for offspring of co-infected females/dams (*n* = 10), but no positive correlation was found. Finally, we tested a model for the prevalence of *Bartonella* and *Babesia* in embryos/pups, but again, no significant association was found.

#### Influence of congenitally acquired *Bartonella* infection on litter size, body mass and survival of pups

Two litters (6 pups of *M. arvalis*, 6 pups of *M. oeconomus*) died 1–2 days after birth. Both *M. arvalis* pups and dam (ID: 2014/107) were *Bartonella*-positive by PCR (Table 4). In the second litter, no positive pups were delivered by the *Bartonella*-positive *M. oeconomus* dam (ID: 2014/74; Fig. 1, Table 4).

All the other pups delivered by 9 *Bartonella*-positive dams (28 *Bartonella*-positive and 22 *Bartonella*-negative pups; Table 4) survived until the end of the experiment. Thus the mortality of pups was 17.6% (6/34; 95% CI: 8.8–31.6%) among *Bartonella*-positive and 21.4% (6/28; 95% CI: 9.8–40.9%) among *Bartonella*-negative pups (ns).

The effect of *Bartonella* infection of the dam on the litter size could not be reliably analyzed as there were no litters from *Bartonella*-negative dams.

We also tested the effect of congenital *Bartonella* infection on the weight of pups at the age of three weeks in a model controlling also for host species and sex. Although the mean weight of *M. oeconomus* pups was significantly higher than for *M. arvalis* pups: 17.56 ± 1.07 and 15.12 ± 0.52 g, respectively (main effect of host species on body mass of pups: *F*_(1, 49)_ = 5.31, *P* = 0.027), no impact of *Bartonella* infection was found (ns). The mean weight was similar for *Bartonella*-positive and *Bartonella*-negative pups (16.68 ± 0.73 and 15.45 ± 0.71g) (ns).

#### Genotyping of *Bartonella* spp. isolates from wild-caught voles and congenitally acquired infections

**Fig. 2:**
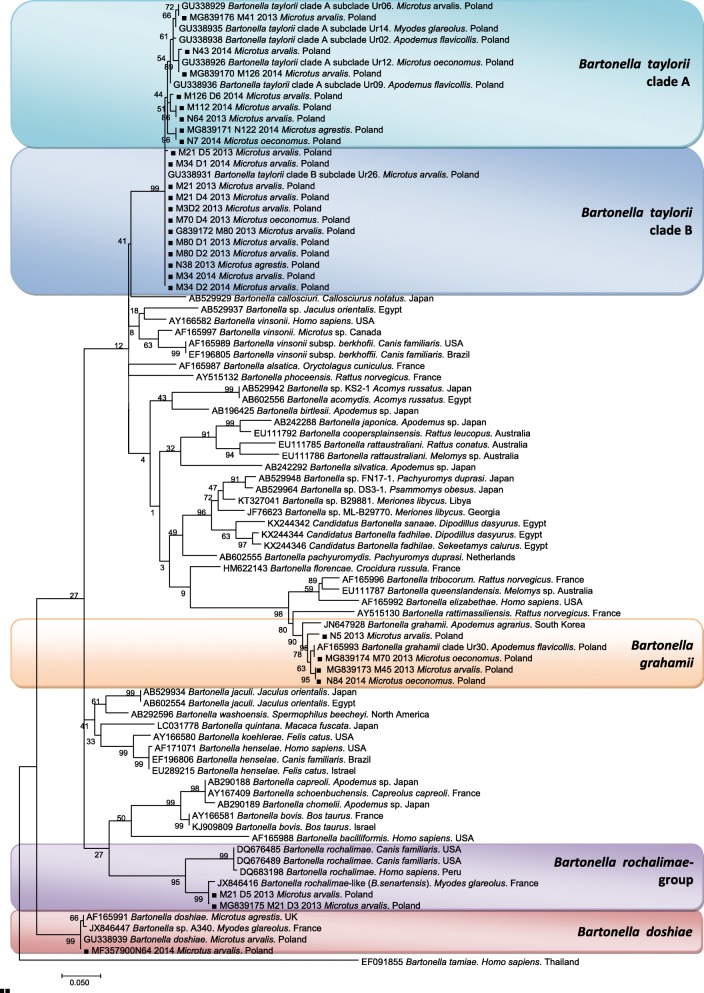
The phylogenetic tree of *Bartonella* spp., based on a fragment of the *rpoB* gene, was inferred using the maximum likelihood method and a Hasegawa-Kishino-Yano (G+I) model. The percentage of replicate trees in which the associated taxa clustered together in the bootstrap test (1000 replicates) are shown next to the branches. The analysis was based on 87 nucleotide sequences. All positions containing gaps and missing data were eliminated. Evolutionary analyses were conducted in MEGA7.0. Black squares indicate the newly generated sequences of the present study

We obtained 113 *Bartonella* sequences from 108 voles (62 adult voles and 46 embryos and pups), including 5 voles for which two different sequences were obtained in independent sequencing events (female nos. 2013/21, 2013/80, 2016/68 and two embryos from the first female: nos. 2013/21/3 and 2013/21/5; Table 3). Altogether 113 (67 *M. arvalis*, 35 *M. oeconomus* and 11 *M. agrestis*) *Bartonella rpoB* sequences were analyzed. Among these, 65 were derived from naturally infected voles, including pregnant females and dams (31 *M. arvalis*, 26 *M. oeconomus* and 8 *M. agrestis*) and 48 were obtained from embryos or pups (36 *M. arvalis*, 9 *M. oeconomus* and 3 *M. agrestis*). Selected representative sequences (including “mother” isolates starting with “M” and embryos/pups isolates marked with mother “M” and offspring “D” numbers) are presented on the phylogenetic tree in Fig. 2 and are deposited in the GenBank database (Additional file 1: Table S1).

Our sequences grouped into four distinct clades representing different species (Fig. 2, Tables 3 and 4). The most common species were *B. taylorii* (89/113, 79%) and then *B. grahamii* (19/113, 17%). Additionally, two sequences of *B. doshiae* were identified. We also identified three sequences of a *B. rochalimae*-like variant (two in embryos and another one from their mother, *M. arvalis*; Table 3).

Among the *B. taylorii* sequences, two main genotypes were found in wild-caught voles, pregnant females and embryos, dams and their pups, both previously described from small rodents from the study area [15]. One genotype was identical (100% homology) with *B. taylorii* clade A (GenBank: GU338926, GU338929) isolated from *M. oeconomus* and *M. arvalis*, respectively, from the Mazury Lake District. The second genotype was most similar (99–100% of homology) to *B. taylorii* clade B (GenBank: GU338928, GU338931), previously isolated from bank vole *M. glareolus* and *M. arvalis*, respectively, from the Mazury Lake District [15] (Fig. 2). Two *B. doshiae* sequences were obtained from wild-caught voles, from *M. arvalis* and *M. oeconomus* displaying highest similarity (98–100%) to *B. doshiae* from *M. arvalis* (GenBank: GU338939) from the study area [15]. A representative sequence of *B. doshiae* has been deposited in GenBank under the accession number MF357900.

Three sequences were obtained (from a *M. arvalis* female and two embryos) which showed highest similarity (98–99%) to a unique *Bartonella* sequence found in *M. glareolus* in France (GenBank: JX846416). These sequences constituted a sister group to the *B. rochalimae* sequences obtained from clinical cases in South and North America. We thus consider them to be a *B. rochalimae*-like variant (Fig. 2); this sequence has been deposited in GenBank under the accession number MG839175.

The *B. taylorii* clade B genotype was dominant among wild-caught voles (32/65, 49%), pregnant females (7/15, 47%) and their embryos (23/27, 85%) and also among dams (6/8, 75%) and their pups (20/21, 95%) (Tables 3 and 4), and altogether 66% of the sequenced isolates corresponded to this genotype.

*Bartonella grahamii* was identified in 17 wild-caught voles (17/65, 26%) (including five pregnant females: 5/15, 33%) and in 7.4% (2/27) of embryos (Tables 3 and 4) but was not found in any of the dams or their pups (Tables 3 and 4), so altogether it was found in 17% of the sequenced isolates.

The *B. taylorii* clade A genotype was identified among wild-caught voles (13/65, 20%), in pregnant females (2/15, 13%) but not in any of their embryos, among dams (2/8, 25%) and in one pup (1/21, 5%) (Tables 3 and 4), and altogether 12% of the sequenced isolates were of this genotype.

Among 65 *Bartonella* sequences obtained from wild-caught voles, 31 were from *M. arvalis*, 26 from *M. oeconomus* and 8 from *M. agrestis*. The *B. taylorii* clade B genotype was identified in 45% of sequences derived from *M. arvalis* (2 from males and 12 from females), in 50% of sequences derived from *M. oeconomus* (5 from males and 8 from females) and in 63% of sequences derived from *M. agrestis* (3 from males and 2 from females).

The *B. taylorii* clade A genotype was identified in 29% of isolates from *M. arvalis* (2 from males and 7 from females), in 4% of sequences derived from *M. oeconomus* (1 from a male) and in three sequences from *M. agrestis* (1 from a male and 2 from females).

*Bartonella grahamii* was identified in 19% of isolates from *M. arvalis* (2 from males and 4 from females) and in 42% of sequences derived from *M. oeconomus* (4 from males and 7 from females).

*Bartonella doshiae* was identified in two female voles (*M. arvalis* and *M. oeconomus-*co-infection with *B. taylorii* clade B) and the *B. rochalimae*-like variant was found in one pregnant *M. arvalis* female (co-infection with *B. taylorii* clade B).

The final step was the correlation of *Bartonella* genotypes infecting mother voles and their corresponding offspring. Sequences of appropriate quality were obtained for 15 pairs. In 12 cases the same species and genotypes were identified in females/dams and their embryos/pups (Tables 3, 4 and Fig. 2: isolates from mothers “Mnn” and offspring “MnnDnn” grouped on the same branches). In one case (female 2013/21) two genotypes (*B. taylorii* clade B and the *B. rochalimae*-like variant) were identified in one individual. In this litter two embryos were co-infected with the *B. rochalimae*-like variant and with *B. taylorii* clade B, but the third embryo was only infected with *B. taylorii* clade B.

Only in three cases were different genotypes found in female-embryo pairs (Table 3). In three cases the *B. taylorii* clade B genotype was isolated from embryos, although *B. grahamii* (2 cases) or *B. taylorii* clade A were identified in their mothers.

Thus, the dominant *Bartonella* genotype identified in offspring was *B. taylorii* clade B, which was found in 85% of 27 embryos and in 95% of 21 pups. There were two other females co-infected concurrently with two *Bartonella* species, but in each of these cases only one of the genotypes was found in the offspring's tissues (females 2013/80 and 2014/68; Table 3).

## Discussion

In the present study, we report a high prevalence of infection with *Bartonella* spp. in a sympatric multi species vole community inhabiting a rural area in north-east Poland. Moreover, we provide evidence that this high prevalence was likely to have been maintained by a significant rate of congenital infections (*via* transplacental transmission from naturally infected female voles to their offspring). Furthermore, our data indicate that vertical transmission of *Bartonella* spp. is unlikely to be associated with the successful vertical transmission of the piroplasm *B. microti*, which was also endemic in these vole populations. Although quite high genetic diversity was identified among the *Bartonella* sequences (4 species comprised of 5 main genetic clades), the *B. taylorii* clade B genotype was the most successful in vertical transmission, being the dominant genotype recovered from *Bartonella* positive embryos and pups.

Our study is among the first to dissect the complex interactions involved in the transmission and circulation of *Bartonella* spp. among a multi-species sympatric *Microtus* vole community. We provide novel data to complement our long-term monitoring of *Bartonella* infections and our earlier work at these same study sites in the Mazury Lake District. The first study on *Bartonella* in voles from this region was carried out in 1997–2000 [33] and focused on *M. arvalis*. Subsequently, in the period 2004–2006, the second study incorporated *M. arvalis* and *M. oeconomus* populations [13, 14], and then between 2007 and 2009 documented the genetic diversity of bartonellae in these vole species [15, 17]. Building on these earlier studies in the present paper, we provide an update on the prevalence of *Bartonella* in more recent years (2013–2014) and focus on the question of why prevalence of *Bartonella* spp. in this community of three vole species (*M. arvalis*, *M. agrestis* and *M. oeconomus*) is consistently so high among the youngest animals which are unlikely to have experienced encounters with hematophagous vectors (and hence unlikely to have been infected after birth).

The overall prevalence of *Bartonella* spp. in this period of 17 years was lowest in the first period (28% in *M. arvalis* [33]). Here we report a much higher prevalence of *Bartonella* spp. (67%) in the *Microtus* community in the period 2013–2014 (73% in *M. arvalis*, 59% in *M. agrestis* and *M. oeconomus*). This much higher prevalence (up to four times higher than previously recorded) can be explained primarily by the fact that sampling was undertaken exclusively in late summer, rather than additionally in the spring when prevalence is generally lower, and by the use of a nested PCR technique for the detection of infection. Seasonal variation in prevalence of *Bartonella* has been reported previously, and prevalence values in late summer and autumn have been found to be at least twice as high as those observed earlier in the year in most studies [7, 23], including in our own earlier work on forest rodents [17, 34]. Nested PCR is a much more sensitive method for the detection of haemoparasites than the single step PCR used earlier, especially in the case of chronic low-level infections [42–44].

It is also possible that this marked increase in the prevalence of *Bartonella* in the *Microtus* community and in each vole species may be attributable to changes in the quality and character of the microhabitat affecting vector populations. Fleas are the main vectors of *Bartonella* spp. among rodents [7, 16, 26] and the life-cycles of these insects includes a free-living larval stage, which is sensitive to unfavourable abiotic conditions, including heavy rain. In 2013, we studied flea infestations in the vole community and we found that the prevalence of fleas was highest among *M. arvalis* (81%) inhabiting the dry upper parts of fallow land, and lowest in root voles (68%) inhabiting the narrow strips surrounding ponds on the grassland, which are often totally submerged during periods of intense rainfall [45]. Additionally, the peak prevalence of *Bartonella* recorded in 2006 was accompanied by the highest recorded infestation by fleas (84% infested rodents in 2006 *versus* 34% in 2004) [13]. We suspected that the exceptionally dry summers in 2013 and 2014 could have created conditions that were particularly suitable for the completion of flea life-cycles and, in turn, this has resulted in higher flea infestations and hence more frequent transmission of bartonellae to voles. Some abiotic data (mean water levels in Poland’s main river, the Vistula River, and largest lake, Lake Śniardwy in the Mazury Lake District) are presented in Additional file 2: Figure S1, and these support the existence of a dry period since 2012. Unfortunately, there is insufficient data yet to test our hypothesis since no data are available on flea infestation in the period 2000–2009, but this is an issue which the continuation of our studies may resolve in the future.

There are few similar studies on *Bartonella* in *Microtus* spp. with which we can directly compare our data. Perhaps the closest was completed on *M. agrestis* in the UK [23] and in this the prevalence of *Bartonella* spp. in autumn (58%) was very similar to that observed in our field voles, although in spring prevalence fell to just over half this level (34%). A similar prevalence was recently found in *M. arvalis* in Slovakia (61% [21]) but in a related host species from the Russian Far East, *Microtus fortis*, prevalence of *Bartonella* was much higher (83% [10]). Overall, most publications report lower prevalence values than those observed here [6, 46–48], but the differences may be the result of the low number of examined voles (5–15 individuals per study) or different season of sampling (most of those studies were conducted in spring-autumn or only in spring). Accordingly, in reported studies DNA extraction was usually performed from tissues rather than blood, which could affect the estimation of *Bartonella* prevalence. Among rodent species studied in the Mazury Lake District, the highest prevalence of *Bartonella* has been previously observed in yellow-necked mice *Apodemus flavicollis* (overall 44–46%, up to 70% in autumn; [13, 49]), followed by the prevalence in bank voles *M. glareolus* (39% [34]).

The main focus of the present study was to test the hypothesis that the perpetuation of *Bartonella* spp. infections in *Microtus* hosts is dependent to some extent on vertical transmission, most likely through the transplacental route as supported by the high fraction of PCR-positive embryos found in this study. This idea was inspired by our consistent observation of a ‘reversed’ age effect in the rodent host, with juvenile individuals more often infected than adults [7, 9, 23]. Indeed, even in the present study this age effect was clearly evident in *M. arvalis*, with the lowest prevalence among mature adults, as in our first study in this area in 1998 and 2000 [33]. Interestingly, in the present study the overall success of vertical transmission of *Bartonella* spp. (calculated collectively for embryos in and pups born to infected mothers) was very similar in all three host species: 50% for *M. arvalis*, 52% for *M. oeconomus* and 50% for *M. agrestis*. Comparing these values with the prevalence of *Bartonella* in juvenile voles (75%, 40% and 100% in *M. arvalis*, *M. oeconomus* and *M. agrestis*, respectively), it is not unreasonable to conclude that the high proportion of *Bartonella* infections recorded among this youngest age class is likely to have been as a consequence of transmission *in utero*, through the placental route and hence congenital or vertical in its nature.

Thus, in addition to the well-established vector-borne route of infection, vertical transmission may also act as an important additional mechanism facilitating the circulation of these bacteria in naturally existing rodent populations. Interestingly, although we noted a high rate of transmission to litters (80–100%) from infected females, not all offspring of infected mothers became infected, with the proportion per litter ranging from 0 to 100% of the embryos/pups becoming infected. The proportion of litters and embryos/pups from infected females that became infected with *Bartonella* in the current study corresponds well with findings in some other studies from different geographical regions. In North America, 80% of litters and 47% of cotton rat (*S. hispidus*) embryos/newborns tested positive for *Bartonella* and 100% of litters and three out of three tested white-footed mouse (*P. leucopus*) embryos were found to be *Bartonella*-positive [27]. In the Barcelona region of Spain, 100% of litters and 69% of embryos of *Bartonella*-positive wood mice (*A. sylvaticus*) were positive [29]. A higher rate of transmission to offspring was recorded in an experimental study in BALB/c mice, with 76% of fetal resorptions positive for *B. birtlesi* [28] but a much lower success of vertical transmission was found in experimentally infected jirds: only one positive pup out of 15 born to infected dams [26].

The success of vertical transmission of *Bartonella* may, therefore, depend to some extent on host species, but there is also evidence that it may depend on the species/genotype of bacteria involved. The pioneering study of Kosoy et al. [27], in which several phylogenetic groups of *Bartonella* were identified by *gltA* gene sequencing, first provided evidence that the efficiency of congenital transmission varied significantly between *Bartonella* genotypes. In the study all *Bartonella* genotypes found in positive embryos/newborns were identical to genotypes in their corresponding mothers, but only two genotypes were successfully transmitted between females and their offspring: genotype A in cotton rats and genotype D in white-footed mice [27]. No positive offspring were found in females infected with genotype C.

We also genotyped *Bartonella* by sequence analysis of a short fragment of the *rpoB* gene, in both females/dams and their corresponding embryos/pups. Four main genetic groups were identified: *B. taylorii* clades A and B, *B. grahamii* and a *B. rochalimae*-like variant. In five litters born to infected dams, no infected offspring were noted, and among these we identified *B. taylorii* clade A (2 litters) and *B. taylorii* clade B (3 cases) (Tables 3 and 4). However, as shown in Table 3, there were four *B. grahamii* infections in females which were not passed on to offspring and only one that was successfully transmitted to offspring. Pertinently, only the *B. taylorii* clade B was identified among positive pups (Table 4) and overall therefore this genotype was the most successful in exploiting the vertical route of transmission. Based on our findings, for *B. grahamii* and the *B. rochalimae*-like variant, vertical transmission is of lesser importance. This conclusion may explain, to some degree, earlier findings of an absence of successful vertical transmission of *Bartonella* in bank voles [12], which are mainly infected with *B. grahamii* [34]. Interestingly, the prevalence of *B. taylorii* in *M. agrestis* in the UK was high in juvenile field voles in autumn, in contrast to *B. doshiae* (which was more prevalent among adults), thus supporting the results of our study [23].

To the best of our knowledge, our study is the first to evaluate the impact of congenital infections of *Bartonella* in wild rodents on the survival and condition of pups. As in the case of congenital infections with *B. microti* [35], we found no evidence for any negative impact: the mortality rate was similar in *Bartonella*-positive and negative pups and the mean weights of infected and uninfected pups were also similar. We compared also mean abundance of infection between wild-caught voles and congenitally infected pups. Contrasting patterns were obtained for the two main host species: in common voles, as in the case of *B. microti* infections, the mean abundance of *Bartonella* spp. was up to ten times higher among adults compared with pups (6.16 iRBC/200 fields *versus* 0.6 iRBC/200 fields). However, in root voles, the pattern was reversed (mean abundance 0.4 iRBC in wild-caught voles *versus* 2.5 iRBC in pups). We found no evidence that the success of vertical transmission of *Bartonella* may be associated with successful vertical transmission of *B. microti*. The exact mechanism of vertical transmission of these pathogens in rodents is not entirely understood; however, in the case of *Bartonella*, bacteria have been found in cultured placental tissues and lesions [27, 28]. Each of these pathogens may have its own specific mechanism for crossing the placental barrier between female and fetus, but the details of this route of transmission remain to be elucidated. Interestingly, in our earlier study in these same animals, the success of vertical transmission of *B. microti* was higher than the success of transmission of *Bartonella* spp., reported here, ranging between 70–81% [35].

Among 113 *Bartonella rpoB* sequences, the most common species was *B. taylorii*, with clade B genotype more frequently detected than clade A, followed by *B. grahamii*. Rare genotypes were those of *B. doshiae* and the *B. rochalimae*-like variant. This pattern is observed among other studies of rodent communities in Europe, in which *B. taylorii* and *B. grahamii* are profoundly dominant in rodents [5–7, 10]. It is also evident that *B. taylorii* is genetically the most variable species, and this may be related to adaptation to different host species [6, 15]). Interestingly, the vast majority of the *rpoB* sequences recorded in the current work were very similar to genotypes obtained from the same rodent community in earlier surveys in 2007–2009, thus confirming their successful circulation in the rodent populations in this region for at least a decade and likely longer [15, 17, 50]. Additionally, in previous studies in this region *B. taylorii* and *B. grahamii* constituted the great majority of identified *Bartonella* species/genotypes [13–15, 17, 34]. In *M. agrestis* in the UK, the distribution of *Bartonella* species/genotypes markedly differed: of the 886 isolates, 264 were *B. doshiae*, 248 *B. grahamii*, 324 *B. taylorii* and 50 of an unidentified *Bartonella* genotype (“BGA”; [23]). This corresponds to an overall prevalence of 15%, 14%, 19% and 3% for *B. doshiae*, *B. grahamii*, *B. taylorii* and the “BGA” variant, respectively. So, although *B. taylorii* was still the most numerous, *B. doshiae* was much more prevalent than in our study.

A relatively rare but distinct genotype that we identified was derived from each of two pups and their mother, which proved to be most similar to *B. rochalimae*. Although *B. rochalimae* is believed to infect rodents [2, 7], our phylogenetic analysis clearly showed that together with a *Bartonella* sequence obtained from a bank vole from France ([6]; a sequence that was very close to ours but not totally identical) our sequences formed a sister branch to *B. rochalimae* isolates from dogs/humans. Buffet et al. [6] proposed a new species name for this genotype, “*Bartonella senartensis*”, but the full description of this species has not yet been completed. Recently in Slovakia, several isolates of *Bartonella* from rodents, including seven sequences from *Microtus* voles, have also been found to be genotypically most similar to *B. rochalimae* [21], but again similarity was relatively low, supporting the existence of a new, rodent-adapted species, that has yet to be comprehensively and formally described.

The final step to complete our study on vertical transmission was to identify and compare the species/genotypes of *Bartonella* in females and their offspring. Although in the majority of female-offspring sets the same genotypes were discovered, the study also revealed the co-infections of two species/genotypes both in females and offspring and successful transmission of only one genotype. We also noticed occurrence of different genotypes in the female-offspring pair (likely due to the above mentioned co-infections in females) and as a result, the occurrence of different genotypes in a single litter. Thus our results show that mixed infections of *Bartonella* species/genotypes can occur in adult voles, with the *B. taylorii* genotype more ‘detectable’ or dominant in adult voles and in offspring. However, the observation that *Bartonella* species/genotypes differ in the success of vertical transmission or in pathogenicity for rodent hosts, requires further study.

## Conclusions

High prevalence of *Bartonella* spp. infection maintained in *Microtus* spp. community is followed by a high rate of vertical transmission of several rodent species of *Bartonella* in three species of naturally infected voles, *M. arvalis*, *M. oeconomus* and *M. agrestis*. Congenitally acquired *Bartonella* infection does not affect the survival of pups. Co-infection with *B. microti* does not affect the effectiveness of the vertical transmission of *Bartonella* in voles. *Bartonella taylorii* clade B was found to be the dominant species in wild-caught voles, including pregnant females and dams, and in their offspring, and was also found to be the most successful in vertical transmission.

## Additional files

Additional file 1: **Table S1.** Accession numbers of representative *Bartonella* spp. 313 bp gene fragment of RNA polymerase β-subunit (*rpoB*) amplified in this study from blood samples from voles near Urwitałt, north-east Poland, in summer 2013 and summer 2014. (DOCX 18 kb)

Additional file 2: **Figure S1.** Mean water level in Lake Śniardwy (**a**) and in the Vistula River (**b**) in the period 1999–2014. (DOCX 17 kb)

## Abbreviations

EDTA: ethylene diamine tetraacetic acid
iRBC: infected red blood cell
ns: not significant
*rpoB*: the *β* subunit of bacterial RNA polymerase
