# Supplementary material for: Bartonella infections in three species of Microtus: prevalence and genetic diversity, vertical transmission and the effect of concurrent Babesia microti infection on its success
Source: Parasit Vectors. 2018 Aug 30;11:491. doi: 10.1186/s13071-018-3047-6 (PMC6117881; doi:10.1186/s13071-018-3047-6)
Supplement: Supplementary file 1 — Table S1. Accession numbers of representative Bartonella spp. 313 bp gene fragment of RNA polymerase β-subunit (rpoB) amplified in this study from blood samples from voles near Urwitałt, north-east Poland, in summer 2013 and summer 2014. (DOCX 18 kb) [file 13071_2018_3047_MOESM1_ESM.docx]

**Table S1.** Accession numbers of representative *Bartonella* spp. 313 bp gene fragment of RNA polymerase β-subunit (*rpoB*) amplified in this study from blood samples from voles near Urwitałt, north-east Poland, in summer 2013 and summer 2014.

| **Accession number** | **Individual number of the sample* (year)** | **Identified species/genotype in the sample**** | **Host species** |
| --- | --- | --- | --- |
| MG839170 | M126 (2014) | *Bartonella taylorii* clade A subclade Ur12 | *M. arvalis* |
| MG839176 | M41(2013) | *Bartonella taylorii* clade A subclade Ur06 | *M. arvalis* |
| MG839171 | N122 (2014) | *Bartonella taylorii* clade B subclade Ur21 | *M. agrestis* |
| MG839172 | M80 (2013) | *Bartonella taylorii* clade B subclade Ur26 | *M. arvalis* |
| MG839173 | M45 (2013) | *Bartonella grahamii* | *M. arvalis* |
| MG839174 | M70 (2013) | *Bartonella grahamii* | *M. oeconomus* |
| MG839175 | M21D3 (2013) | *Bartonella rochalimae*-like | *M. arvalis* |
| MF357900 | N64 (2014) | *Bartonella doshiae* | *M. arvalis* |

* Number starting with “M”: number of mother/dam; number starting with “M” and containing “D”:, number of the pup/embryo including its mother number; number starting with “N”:, number of wild-caught voles (not involved in laboratory breeding colony).

**Following the nomenclature by Paziewska et al.

**References**

Paziewska A, Harris PD, Zwolinska L, Bajer A, Sinski E. Recombination within and between species of the alpha proteobacterium *Bartonella* infecting rodents. Microb Ecol. 2011;61:134–45.

Paziewska A. Diversity of blood parasites of genus *Bartonella* in wild rodents in Mazury Lakes District. PhD thesis, University of Warsaw, Warsaw, Poland; 2010 (In Polish).
