# Supplementary material for: Bartonella infections in three species of Microtus: prevalence and genetic diversity, vertical transmission and the effect of concurrent Babesia microti infection on its success
Source: Parasit Vectors. 2018 Aug 30;11:491. doi: 10.1186/s13071-018-3047-6 (PMC6117881; doi:10.1186/s13071-018-3047-6)
Supplement: Supplementary file 2 — Figure S1. Mean water level in Lake Śniardwy (a) and in the Vistula River (b) in the period 1999–2014. (DOCX 17 kb) [file 13071_2018_3047_MOESM2_ESM.docx]

**Additional file 2: Figure S1.** Mean water level in Lake Śniardwy (**a**) and in the Vistula River (**b**) in the period 1999–2014.
